# Supplementary material for: Suppressive IL-17A+Foxp3+ and ex-Th17 IL-17AnegFoxp3+ Treg cells are a source of tumour-associated Treg cells
Source: Nat Commun. 2017 Mar 14;8:14649. doi: 10.1038/ncomms14649 (PMC5355894; doi:10.1038/ncomms14649)
Supplement: Supplementary Information — Supplementary Figures and Supplementary Tables [file ncomms14649-s1.pdf]

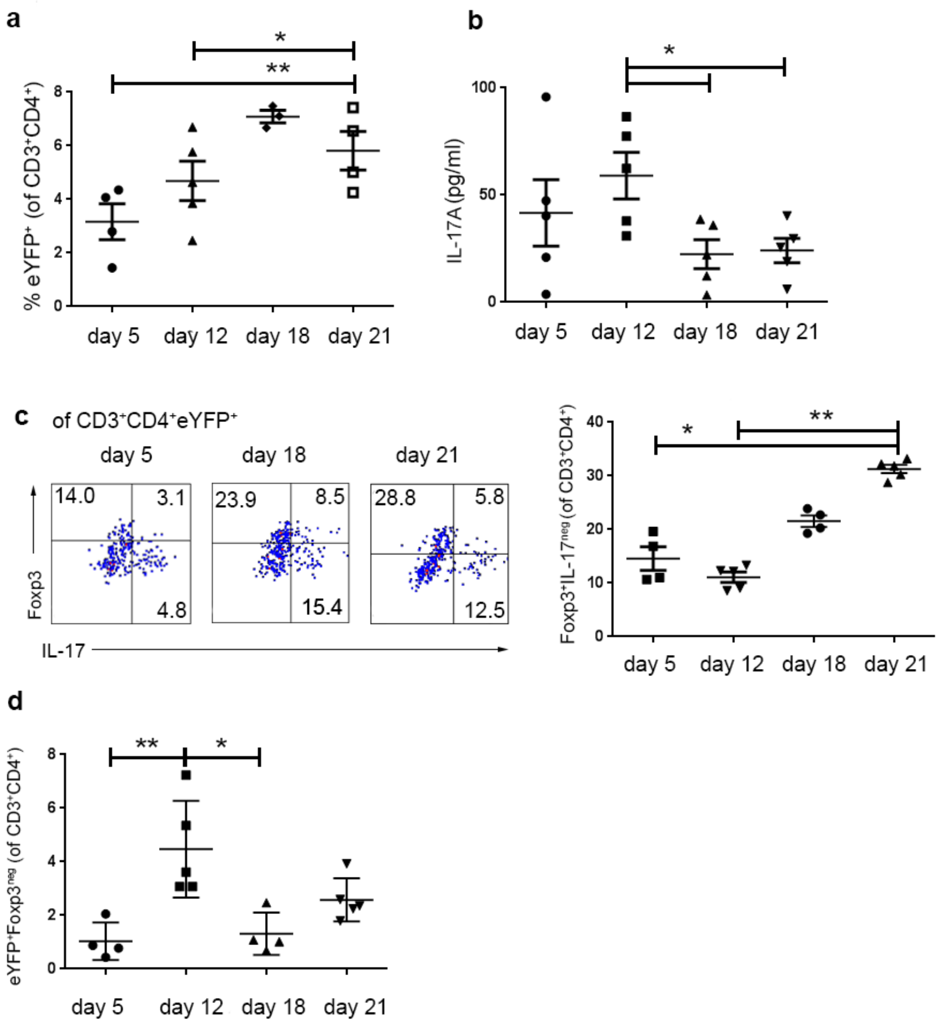

Supplementary Figure 1. Th17 cells transdifferentiate into *Foxp3*$^{+}$ *exTh17* cells in tumor-bearing mice. MC38 cells were injected in IL17a^{Cre}R26R^{ReYFP} fate reporter mice. Splenocytes were recovered at different time points (*n = 4 mice per group*) of tumor progression and CD4^{+} T cells assessed for eYFP expression (**a**), IL-17 production (**b**) and eYFP^{+} cells analyzed for IL-17 production and Foxp3 expression (**c,d**) by flow cytometry. (**a**) We observed accumulation of eYFP^{+} cells (indicative of Th17 and/or exTh17 cells) over time. (**b**) IL-17 production, as determined by ELISA, showed a decline in IL-17 cytokine after the initial induction. (**c**) The percentages of Foxp3^{+}IL-17^{neg} (i.e. exTh17 T$_{reg}$) cells increased in these mice with tumor progression, which matched with the reduction of the percentage of eYFP^{+} cells not expressing Foxp3 (**c**). All data are mean ± s.d. *$P < 0.05$ and **$P < 0.01$ by two-tailed Student's *t* test. Similar results were obtained in an additional independent experiment.

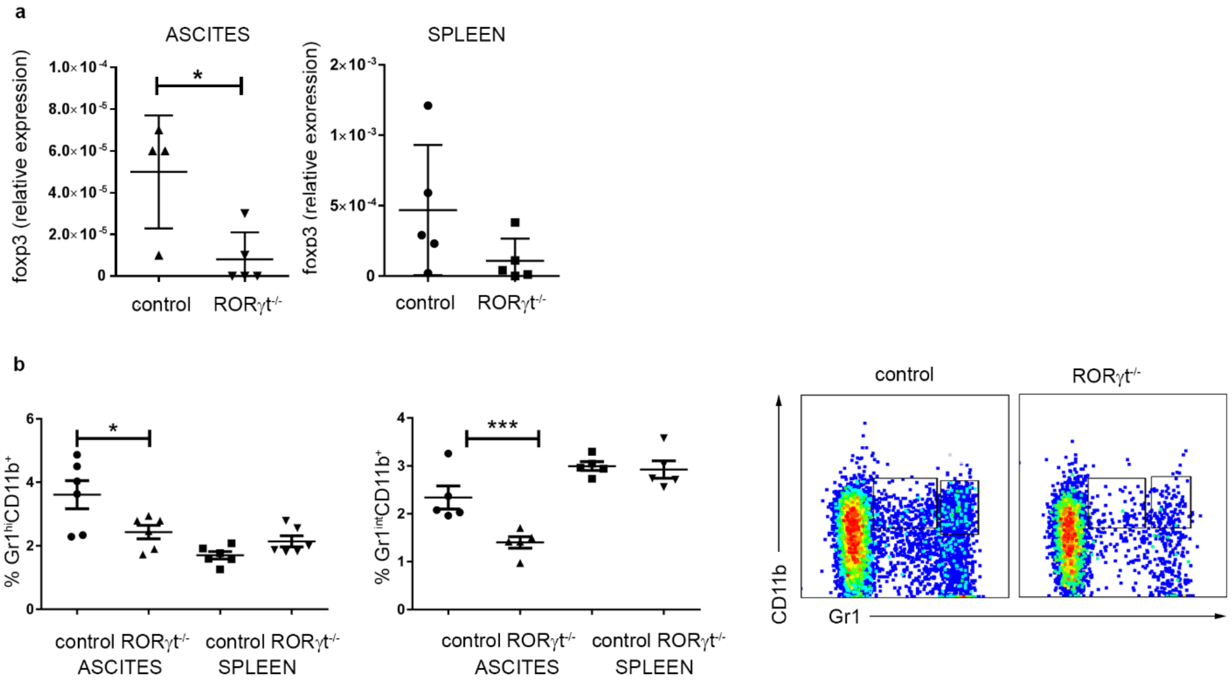

Supplementary Figure 2. RORγt$^{-/-}$ mice demonstrate reduced infiltration of Foxp3^{+} T$_{reg}$ cells and MDSCs in tumor microenvironment. ID8 cells (4x10^{6} per mouse) were injected in RORgt$^{-/-}$ mice. Ascites-infiltrating cells and splenocytes were isolated on day 35±2 (*n = 5 mice per group*) of tumor progression (**a**) Foxp3 expression in the TALs and spleens was determined on day 35±2. (**b**) Statistical analysis (*left*) and the representative staining (*right*) of Gr1$^{hi/int}$CD11b^{+} cells in the tumor ascites and spleens from $n = 5$ mice. All data are mean ± s.d. *$P < 0.05$, **$P < 0.01$ by two-tailed Student's t test.

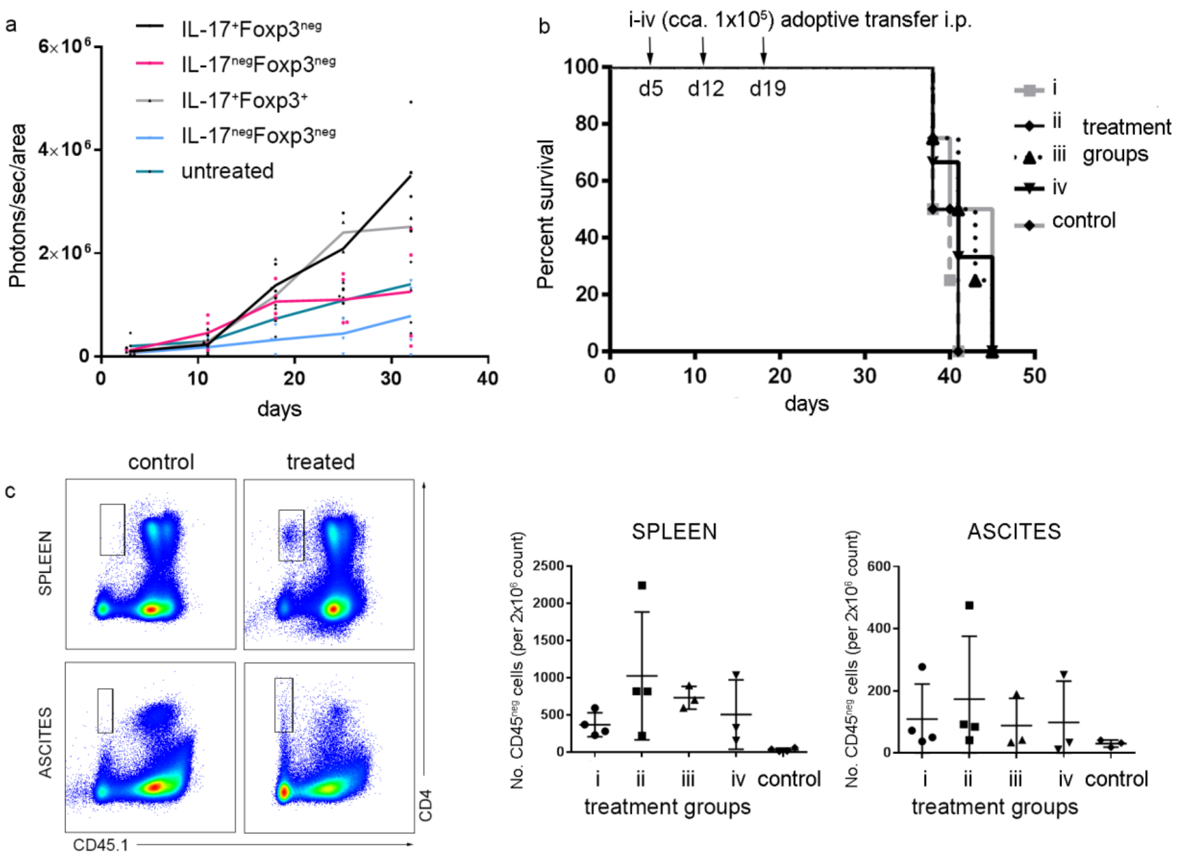

Supplementary Figure 3. Adoptively transfered Foxp3$^{neg/+}$IL-17$^{neg/+}$ T cells do not significantly alter the survival of ID8A tumor-bearing mice. CD4^{+} T cells from *Foxp3^{GFP}* reporter mice were cultured under Th17 (IL-6, IL-23, TGF-β, 3 days)/T$_{reg}$ (TGF-β, +1 day) -driving conditions. Foxp3^{neg}IL-17^{+} (i), Foxp3^{+}IL-17^{+} (ii), Foxp3^{neg}IL-17^{neg} (iii) and Foxp3^{+}IL-17^{neg} (iv) CD3^{+} T cells were sorted and injected i.p. into ID8-tumor bearing mice as outlined in **Fig. 3a**. (**a**,**b**) Th17-T$_{reg}$ subsets (i-iv) were injected i.p. into ID8A-luc tumor-bearing Cd45.1 mice on days 5, 12 and 19 and the mice monitored for tumor progression (**a**) and survival (**b**, weight increase >140%, lethargic behavior). (**c**) CD4^{+}CD45.1^{neg} cells from ID8A-luc tumor-bearing Cd45.1 mice, receving adoptively transfered Th17-T$_{reg}$ subsets (groups i-iv, *n = 4* mice per group), were analyzed on day 40±1. Representative staining (*left*) and the numbers of CD4^{+}CD45.1^{neg} cells (*right*) in each group of mice.

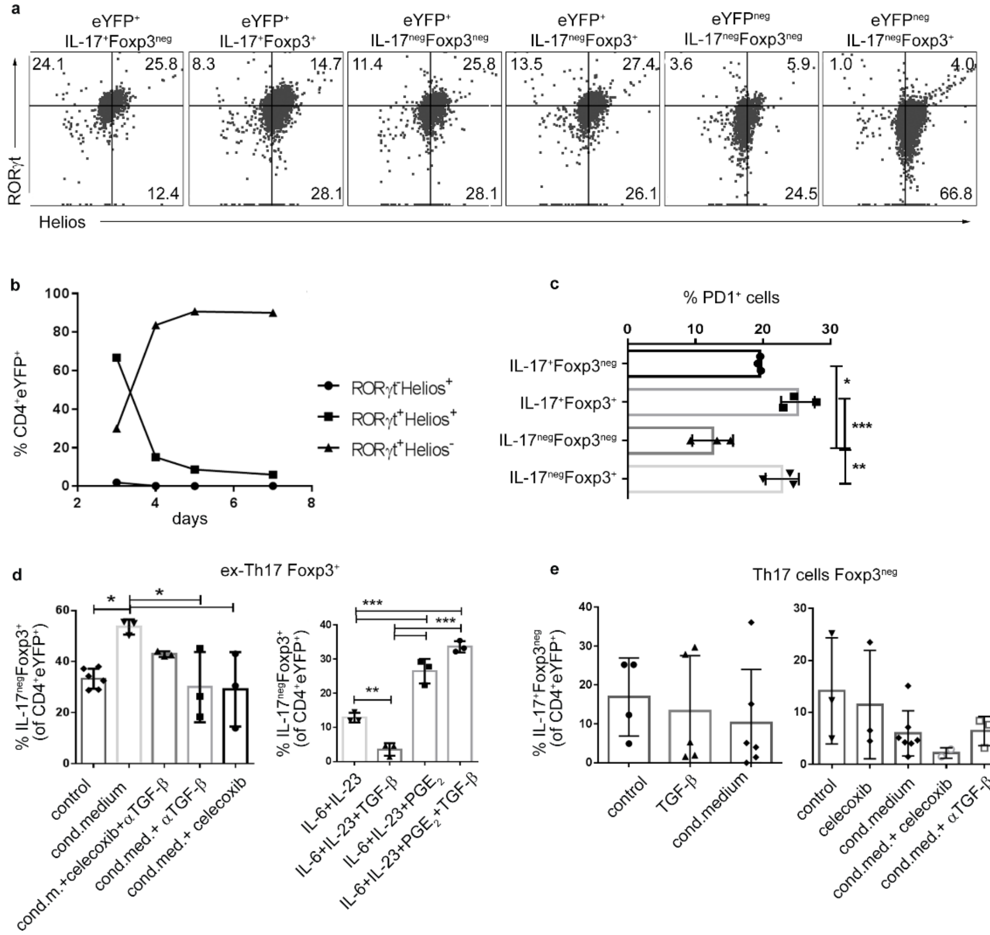

Supplementary Figure 4. Characteristics of Th17 into ex-Th17 Foxp3^{+}IL-17^{neg} cells-transdifferentiation. (**a**) YFP^{+}Foxp3^{neg}IL-17^{+}, YFP^{+}Foxp3^{+}IL-17^{+}, YFP^{+}Foxp3^{neg}IL-17^{neg}, YFP^{+}Foxp3^{+}IL-17^{neg} and YFP^{neg}Foxp3^{neg}IL-17^{neg}, YFP^{+}Foxp3^{neg}IL-17^{neg} CD3^{+} T cells were sorted using the strategy presented in **Fig. 4e** and analyzed for RORγt and Helios expression by flow cytometry. Similar data was obtained in an additional independent experiments. (**b**) Representative flow cytometry analysis of time-dependent induction of Helios and RORγt in eYFP^{+} cells from Il17a^{Cre}R26R^{eYFP} fate reporter mice (CD4^{+} gated). Cells were cultured in the presence of Th17 (IL-6, IL-23 and TGF-β)- driving conditions and analyzed on days 3, 4, 5 and 7 by flow cytometry for the expression of Helios and RORγt by eYFP^{+} cells. Similar data was obtained in an additional independent experiments. (**c**) Foxp3^{neg}IL-17^{+}, Foxp3^{+}IL-17^{+}, Foxp3^{neg}IL-17^{neg} and Foxp3^{+}IL-17^{neg} CD3^{+} T cells were sorted using the strategy presented in **Fig. 3a** and analyzed by flow cytometry for PD1 expression. Aggregate data of 3 independent experiments is shown as mean ± sd. (**d**) CD4^{+} T cells from *IL-17a^{Cre}R26R^{ReYFP}* reporter mice were cultured in conditioned medium of ID8A cells (mean ± sd, *left*) and analyzed for the percentage of IL-17^{neg}Foxp3^{+} cells of YFP^{+}CD4^{+} cells. COX2 inhibitor celecoxib was added during preparation of conditioned medium and celecoxib and TGF-β blocking antibody were added to cell cultures during CD3/CD28 stimulation. CD4^{+} T cells from IL-17a^{Cre}R26R^{ReYFP} reporter mice were cultured in the presence of cytokines IL-6, IL-23, TGF-β and PGE_2 as indicated (mean ± sd, *right*). Similar data was obtained in an additional independent experiments. (**e**) CD4^{+} T cells from IL-17a^{Cre}R26R^{ReYFP} reporter mice were cultured under T_{reg}-driving conditions (TGF-β or conditioned medium of ID8A cells) and analyzed for the percentage of IL-17^{+}Foxp3^{neg} cells of YFP^{+}CD4^{+} cells (mean ± sd, *left*). COX2 inhibitor celecoxib was added during preparation of conditioned medium and celecoxib and TGF-β blocking antibody

were added to cell cultures during CD3/CD28 stimulation, *right*). Similar data was obtained in an additional independent experiments. *$P < 0.05$, **$P < 0.01$ and ***$P < 0.001$ by one-way ANOVA.

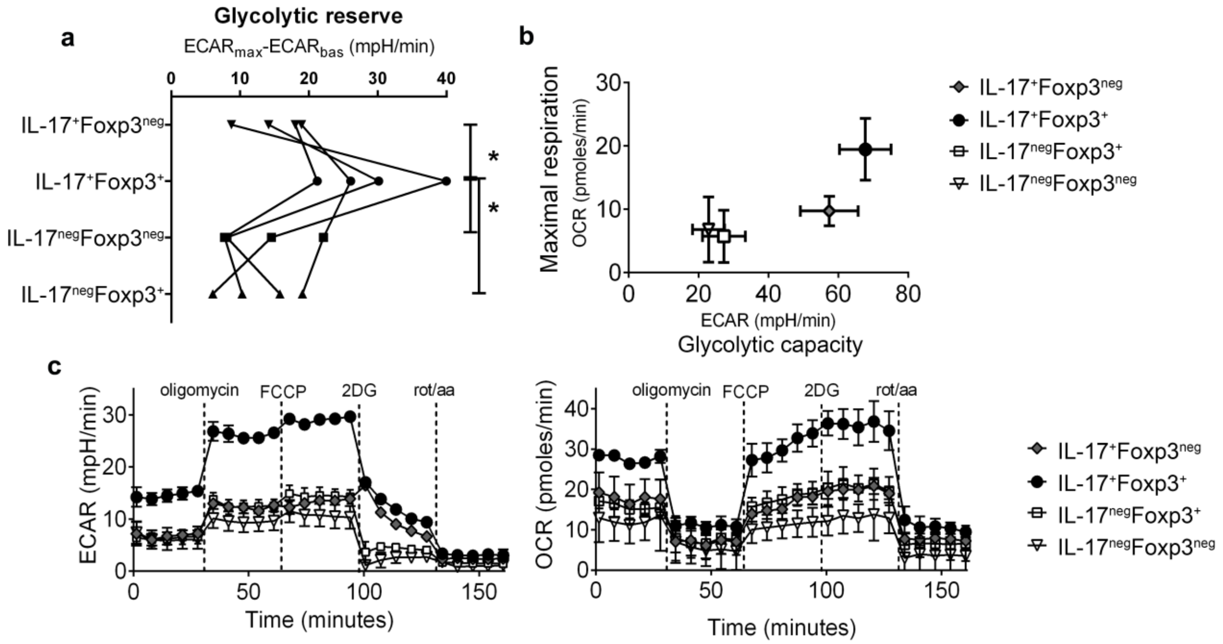

Supplementary Figure 5. High metabolic activity of immunosuppressive Foxp3^{+}IL-17^{+} cells. (**a-c**) Foxp3^{neg}IL-17^{+}, Foxp3^{+}IL-17^{+}, Foxp3^{neg}IL-17^{neg} and Foxp3^{+}IL-17^{neg} CD3^{+} T cells were sorted using the strategy presented in **Fig. 4e** and analyzed with XF^{e} Extracellular Flux Analyzer. (**a**) Glycolytic reserve is defined as the difference between the mean basal ($ECAR_{bas}$) and maximal ECAR ($ECAR_{max}$) values. Mean of each real-time run for individual subset was calculated. Cumulative data from 4 independent experiments are evaluated by one-way ANOVA, $*P < 0.05$. (**b**) Representative data of maximal respiration vs glycolytic capacity of individual Th17-T$_{reg}$ subsets (*n = 4* Foxp3^{+}IL-17^{+}, *n = 8* Foxp3^{+}IL-17^{neg}, *n = 6* Foxp3^{neg}IL-17^{+}, *n = 4* Foxp3^{neg}IL-17^{neg}). (**c**) Real-time measurements (mean ± sd) of glycolysis (ECAR, *left*) and oxidative phosphorylation (OCR, *right*) in 96-well Seahorse assay plates (approx. 1×10^{5} cell/well) were carried out as described in Online Methods. Graphs are representative of three independent experiments and show the effects of oligomycin, 2DG, mitochondrial inhibitors FCCP and rotenone, injected as indicated.

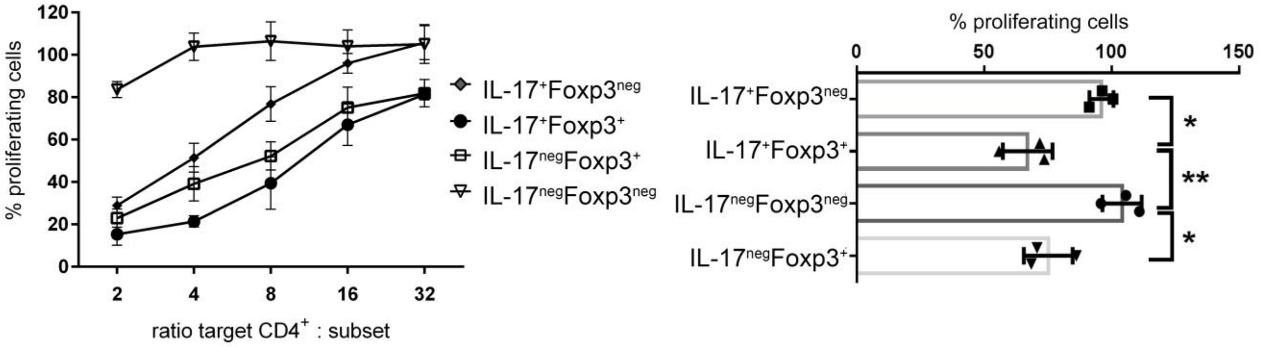

Supplementary Figure 6. High immunosuppressive activity of IL-17^{+}Foxp3^{+} cells. Foxp3^{neg}IL-17^{+}, Foxp3^{+}IL-17^{+}, Foxp3^{neg}IL-17^{neg} and Foxp3^{+}IL-17^{neg} CD3^{+} Th17-T$_{reg}$ subsets were sorted using the strategy presented in **Fig. 3a**. CD4^{+} T cells, isolated from Cd45.1 mice and stained with CFSE, were analyzed after 72h stimulation with αCD3Ab, in the presence of irradiated CD4^{neg} fraction and different ratios of Th17-T$_{reg}$ subsets. One-way ANOVA of immunosuppressive effects of the plasticity subsets at 1:4 ratio subset:CD4^{+} (*right*).

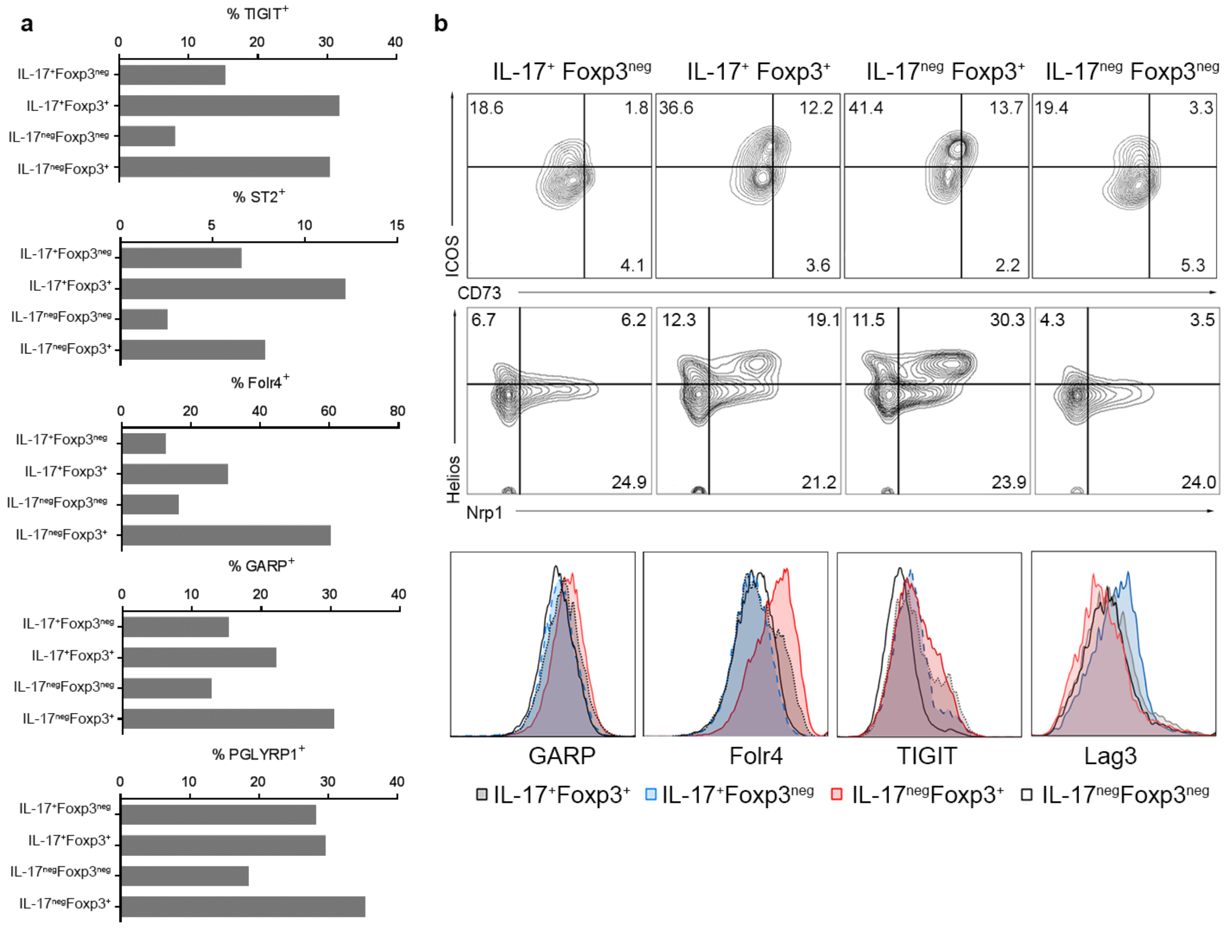

Supplementary Figure 7. Immunophenotyping of IL-17^{+}Foxp3$^{+/neg}$ cells. (a,b) Foxp3^{neg}IL-17^{+}, Foxp3^{+}IL-17^{+}, Foxp3^{neg}IL-17^{neg} and Foxp3^{+}IL-17^{neg} CD3^{+} T cells were sorted using the strategy presented in **Fig. 3a**, stained for respective Th17-T$_{reg}$ markers and analyzed by flow cytometry. Percentages of TIGIT^{+}, ST2^{+}, Folr4^{+}, GARP^{+} and PGLYRP1^{+} cells (**a**) and representative flow cytometry staining (**b**) of each Th17-T$_{reg}$ subset. Similar data was obtained in an additional independent experiments.

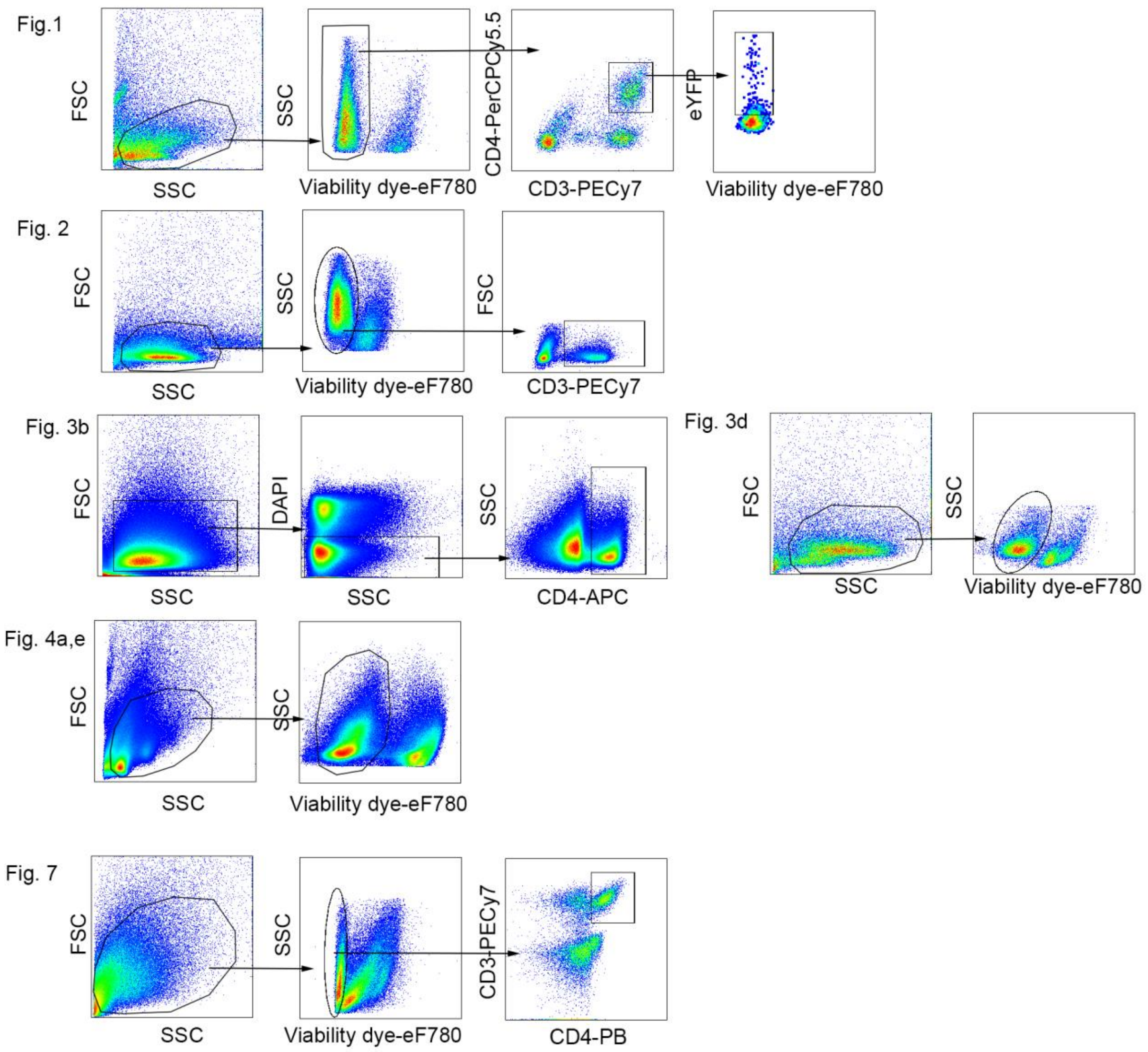

Supplementary Figure 8. Gating strategy. The FACS gating strategy of live eYFP$^+$ CD4$^+$ T cells presented in **Fig. 1** (**a**), live T cells presented in **Fig. 2** (**b**), live CD4$^+$ cells presented in **Fig. 3b** (**c**), live cells presented in **Fig. 3d** (**d**), live cells presented in **Fig. 4a,e** (**e**) and live CD4$^+$ T cells presented in **Fig. 7** (**f**).

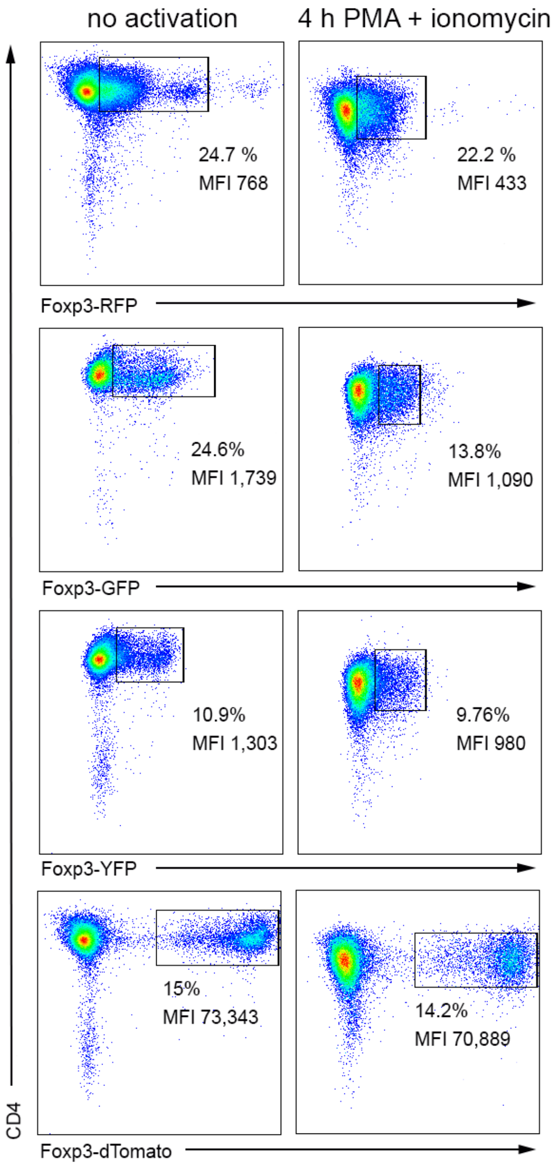

Supplementary Figure 9. Activation with PMA and ionomycin prior to cell sorting results in a reduced mean fluorescence intensity of Foxp3 reporter signal. $CD4^+$ cells were activated with anti-CD3/CD28 microbeads in the presence of irradiated $CD4^{neg}$ fraction and 5ng/ml TGF-β and analyzed by flow cytometry with or without prior 4 h stimulation with PMA and ionomycin. The activated cells have lower mean fluorescence intensity of the Foxp3 reporter protein mRFP ($Foxp3^{mRFP}$ mice), GFP ($Foxp3^{GFP}$ mice), YFP and dTomato ($Foxp3^{YFP/Cre}Rosa26^{tdTomato}$ mice), respectively) following stimulation with PMA and ionomycin. Similar data was obtained in an additional independent experiments.

Transcript Cluster ID	IL17^{+}Foxp3^{neg} Bi-weight Avg Signal (log2)	IL17^{+}Foxp3^{+} Bi-weight Avg Signal (log2)	Fold Change (linear)	ANOVA p-value	FDR p-value	Gene Symbol	Group
TC0X00000058.mm.1	5.32	10.35	-32.83	0.013334	0.815157	Foxp3	Complex
TC0900001779.mm.1	8.02	12.2	-18.2	0.013635	0.815157	Folr4	Coding
TC0700001368.mm.1	5.8	9.88	-16.96	0.010621	0.815157	Lrrc32	Coding
TC0900000113.mm.1	5.28	9.21	-15.26	0.023053	0.815157	Gpr83	Complex
TC0100002534.mm.1	7.72	10.92	-9.17	0.000966	0.815157	Ikzf2	Complex
TC1200002511.mm.1	4.65	7.79	-8.81	0.049593	0.822847	Itgb8	Complex
TC0200001018.mm.1	6.55	9.2	-6.29	0.045543	0.822847	Myo3b	Complex
TC0100000298.mm.1	5.05	7.48	-5.38	0.03197	0.815157	Il1rl1	Coding
TC0700000275.mm.1	6.23	8.66	-5.38	0.016924	0.815157	Pglyrp1	Coding
TC0300000656.mm.1	8.01	10.3	-4.87	0.020658	0.815157	Tmem154	Complex
TC1100001015.mm.1	10.58	12.81	-4.7	0.021584	0.815157	Itgae	Coding
TC1600001633.mm.1	9.78	11.85	-4.21	0.012566	0.815157	Tigit	Coding
TC1200002542.mm.1	14.19	16.1	-3.77	0.010202	0.815157	Ighm	Complex
TC0600003547.mm.1	3.65	5.49	-3.57	0.010338	0.815157	Igkv4-91	Coding
TC1600001580.mm.1	7.79	9.59	-3.47	0.044093	0.822847	Cd86	Complex
TC0800002310.mm.1	5.09	6.77	-3.2	0.015858	0.815157	Cpe	Complex
TC0200003532.mm.1	12.76	14.38	-3.09	0.040506	0.822847	Arl5a	Complex
TC1900000572.mm.1	7.32	8.91	-3	0.031331	0.815157	Entpd1	Complex
TC1000002099.mm.1	8.32	9.88	-2.96	0.019111	0.815157	Prdm1	Complex
TC0100000490.mm.1	14.17	15.71	-2.91	0.018253	0.815157	Icos	Coding
TC0100000216.mm.1	5.71	7.22	-2.85	0.02423	0.815157	Dst	Complex
TC0100003589.mm.1	7.06	8.55	-2.8	0.039297	0.822847	Gm4955	Coding
TC1600001324.mm.1	5.46	6.92	-2.76	0.038858	0.822847	Iglv1	Complex
TC0900001675.mm.1	9.36	10.81	-2.74	0.045643	0.822847	Ccr5	Complex
TC0400003957.mm.1	9.91	11.32	-2.65	0.001322	0.815157	Tnfrsf1b	Complex
TC1100003345.mm.1	5.45	6.86	-2.65	0.031189	0.815157	Myo1d	Complex
TC0800002804.mm.1	11.01	12.4	-2.61	0.034603	0.815157		Other
TC1200002626.mm.1	3.38	4.73	-2.55	0.035149	0.815157	Ighv1-47	Coding
TC0500002855.mm.1	9.43	10.77	-2.53	0.027131	0.815157	LOC636711	Coding
TC1100003680.mm.1	10.85	12.17	-2.51	0.039594	0.822847	Ikzf3	Complex
TC0500000934.mm.1	10.17	11.46	-2.45	0.011954	0.815157	Bmp2k	Complex
TC0100002844.mm.1	7.56	8.79	-2.35	0.017411	0.815157	St8sia4	Coding
TC0700000995.mm.1	10.39	11.57	-2.28	0.049193	0.822847	Gm10974	Coding
TC1700000044.mm.1	8.69	9.85	-2.24	0.034704	0.815157	Snx9	Complex
TC0300000680.mm.1	8.94	10.06	-2.18	0.025938	0.815157	Lrba	Complex
TC0300002123.mm.1	11.8	12.93	-2.18	0.005385	0.815157	Gm20689	Complex
TC1200002606.mm.1	4.26	5.34	-2.12	0.019281	0.815157	Ighv1-11	Unassigned
TC0800000653.mm.1	6.46	7.54	-2.11	0.014936	0.815157	Sh3rf1	Coding
TC1000001691.mm.1	9.24	10.29	-2.08	0.046896	0.822847	Ipcef1	Complex
TC1200002573.mm.1	5.74	6.79	-2.08	0.015612	0.815157	Ighv2-9	Coding
TC1400002781.mm.1	8.69	9.69	-2.01	0.043903	0.822847	Gm10340	Complex

Supplementary Table 1. Differentially expressed genes in IL17^{+}Foxp3^{neg} and IL17^{+}Foxp3^{+} cells. The list of genes upregulated in IL17^{+}Foxp3^{+} compared to IL17^{+}Foxp3^{neg} cells. The filters applied were ANOVA $p<0.05$ and linear fold change <-2. The non-coding genes were excluded.

Transcript Cluster ID	IL17^{+}Foxp3^{+} Bi-weight Avg Signal (log2)	IL17^{neg}Foxp3^{+} Bi-weight Avg Signal (log2)	Fold Change (linear)	ANOVA p-value	FDR p-value	Gene Symbol	Group
TC0600003606.mm.1	4.53	7.36	-7.1	0.068843	0.932327	Klrb1b	Coding
TC1400000799.mm.1	4.1	6.35	-4.75	0.020511	0.932327	Mcpt1	Coding
TC0300001616.mm.1	5.63	7.59	-3.9	0.083915	0.932327	Hey1	Coding
TC1500001828.mm.1	5.24	7.07	-3.57	0.01557	0.932327	Csf2rb2	Complex
TC1600000553.mm.1	8.28	10.09	-3.51	0.082335	0.932327	Cd80	Complex
TC1300000144.mm.1	7.76	9.56	-3.48	0.014222	0.932327	Tcrg-C3	Coding
TC1300002762.mm.1	8.79	10.51	-3.28	0.010034	0.932327	Tcrg-V4	Complex
TC1400000040.mm.1	9.7	11.26	-2.94	0.035491	0.932327	Gm3317	Complex
TC0800000676.mm.1	8.96	10.48	-2.89	0.027532	0.932327	Gm10663	Coding
TC1400000030.mm.1	9.09	10.58	-2.79	0.061513	0.932327	Gm3239	Coding
TC0700002139.mm.1	3.85	5.28	-2.69	0.013411	0.932327	Gm15925	Pseudogene
TC0900000720.mm.1	7.72	9.14	-2.68	0.014858	0.932327	Sema7a	Complex
TC0900000240.mm.1	9.8	11.17	-2.59	0.077555	0.932327	Gm10181	Coding
TC1000001786.mm.1	4.71	6.07	-2.56	0.078135	0.932327	Gpr126	Complex
TC0100002552.mm.1	4.19	5.52	-2.51	0.038213	0.932327	Mreg	Coding
TC1500000641.mm.1	5.11	6.43	-2.5	0.001263	0.863213	Csf2rb	Complex
TC1100003097.mm.1	5.18	6.49	-2.48	0.01681	0.932327	Cxcl16	Complex
TC1300000364.mm.1	5.12	6.43	-2.48	0.0425	0.932327	Serpinb9	Coding
TC1200001660.mm.1	4.09	5.36	-2.41	0.046271	0.932327	Scin	Coding
TC0100000509.mm.1	4.55	5.78	-2.36	0.077042	0.932327	Nrp2	Complex
TC0700004632.mm.1	4.42	5.63	-2.32	0.064539	0.932327	Pira1	Complex
TC1200002572.mm.1	4.51	5.71	-2.3	0.009091	0.932327	Ighv5-17	Coding
TC0300000807.mm.1	7.54	8.74	-2.3	0.069762	0.932327	S100a4	Complex
TC0600001369.mm.1	7.48	8.69	-2.3	0.065269	0.932327	Usp18	Coding
TC1400002851.mm.1	9.18	10.37	-2.28	0.072272	0.932327	Gm3739	Complex
TC0700004634.mm.1	4.42	5.6	-2.27	0.055745	0.932327	Gm14548	Coding
TC1400002845.mm.1	9.61	10.77	-2.24	0.046524	0.932327	Gm3629	Coding
TC0700002087.mm.1	10.86	12.01	-2.22	0.070881	0.932327	Tspan32	Complex
TC1000001625.mm.1	7.49	8.63	-2.21	0.06409	0.932327	Cd63	Coding
TC0300002482.mm.1	6.95	8.09	-2.2	0.0223	0.932327	Ecm1	Complex
TC0900001048.mm.1	5.2	6.33	-2.19	0.041569	0.932327	Gsta4	Complex
TC1800000510.mm.1	7.88	9.01	-2.19	0.020736	0.932327	Cd63-ps	Pseudogene
TC1400000046.mm.1	9.83	10.95	-2.17	0.075524	0.932327	Gm3488	Complex
TC0500000928.mm.1	4.78	5.85	-2.11	0.024857	0.932327	Anxa3	Coding
TC1600000479.mm.1	5.59	6.62	-2.04	0.074893	0.932327	Muc13	Coding
TC0900001824.mm.1	3.45	4.49	-2.04	0.091642	0.932327	Olfr860	Coding
TC1500000982.mm.1	7.18	8.2	-2.03	0.047668	0.932327	Cacnb3	Coding
TC0500000843.mm.1	4.77	5.79	-2.03	0.075706	0.932327	Pf4	Coding
TC1400002792.mm.1	10.06	11.07	-2.02	0.090881	0.932327	Gm3252	Coding
TC0700003485.mm.1	8.53	7.52	2.02	0.039077	0.932327	Mctp2	Complex
TC0X00002316.mm.1	8.39	7.36	2.03	0.057587	0.932327	Gm8163	Pseudogene
TC1000001141.mm.1	10.09	8.99	2.15	0.039393	0.932327	Gm3571	Pseudogene
TC0500003479.mm.1	8.73	7.62	2.15	0.059527	0.932327	Got2-ps1	Pseudogene
TC1900001510.mm.1	9.25	8.08	2.25	0.036734	0.932327	Arhgap19	Coding
TC0X00002996.mm.1	5.25	4.07	2.27	0.040605	0.932327	Gm7855	Pseudogene
TC0800000424.mm.1	7.25	5.94	2.49	0.081573	0.932327	Ppp1r3b	Complex

Supplementary Table 2. Differentially expressed genes in IL17^{+}Foxp3^{+} and IL17^{neg}Foxp3^{+} cells. The list of genes upregulated in IL17^{neg}Foxp3^{+} compared to IL17^{+}Foxp3^{+} cells. The filters applied were ANOVA $p<0.05$ and linear fold change <-2. The non-coding genes were excluded.
